# Ouabain suppresses the growth and migration abilities of glioma U-87MG cells through inhibiting the Akt/mTOR signaling pathway and downregulating the expression of HIF-1α

**DOI:** 10.3892/mmr.2021.11836

**Published:** 2021-01-11

**Authors:** Xiao-Sa Yang, Zhong-Wei Xu, Tai-Long Yi, Rui-Cheng Xu, Jie Li, Wen-Bin Zhang, Sai Zhang, Hong-Tao Sun, Ze-Qi Yu, Hao-Xiang Xu, Yue Tu, Shi-Xiang Cheng

Mol Med Rep 17: 5595-5600, 2018; DOI: 10.3892/mmr.2018.8587

Subsequently to the publication of the above paper, an interested reader drew to the authors' attention that several pairings of panels in Fig. 5, as shown on p. 5599, were strikingly similar. After having examined their original data, the authors realized that they uploaded some images incorrectly during the process of compiling this figure, and that there were duplicated data panels in this figure. However, the authors were able to consult their original data, and had access to the correct images.

The revised version of Fig. 5, showing the correct data for the Akt/Control, p-Akt/Control, mTOR/0.05 μM Ouabain, HIF-1α/0.05 μM Ouabain and Akt/0.5 μM Ouabain experiments, is shown opposite. Note that the replacement of the erroneous data does not affect either the results or the conclusions reported in this paper, and all the authors agree to this Corrigendum. The authors are grateful to the Editor of *Molecular Medicine Reports* for granting them this opportunity to publish a Corrigendum, and apologize to the readership for any inconvenience caused.

## Figures and Tables

**Figure 5.**
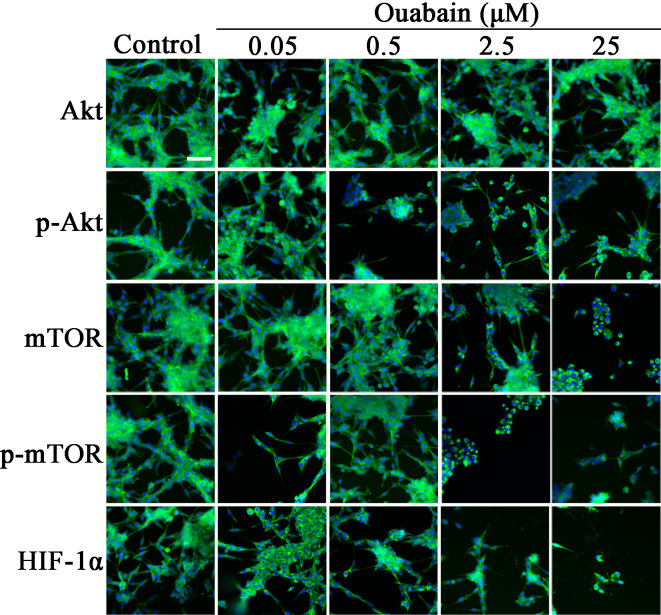
High content screening analyses of the changes in protein levels treated with ouabain. U-87MG cells were treated with the different concentrations of ouabain for 24 h and fluorescence images were captured. Scale bar=50 µm. mTOR, mammalian target of rapamycin; p-, phosphorylated; HIF-1α, hypoxia inducible factor-1α.

